# A Framework for Adapting Deep Brain Stimulation Using Parkinsonian State Estimates

**DOI:** 10.3389/fnins.2020.00499

**Published:** 2020-05-19

**Authors:** Ameer Mohammed, Richard Bayford, Andreas Demosthenous

**Affiliations:** ^1^Department of Electronic and Electrical Engineering, University College London, London, United Kingdom; ^2^Department of Mechatronic Engineering, Air Force Institute of Technology, Kaduna, Nigeria; ^3^Department of Natural Sciences, Middlesex University, London, United Kingdom

**Keywords:** biomedical signal processing, deep brain stimulation (DBS), feature extraction, fuzzy control, Gaussian mixture models, support vector machine, Parkinson's disease, state estimator

## Abstract

The mechanisms underlying the beneficial effects of deep brain stimulation (DBS) for Parkinson's disease (PD) remain poorly understood and are still under debate. This has hindered the development of adaptive DBS (aDBS). For further progress in aDBS, more insight into the dynamics of PD is needed, which can be obtained using machine learning models. This study presents an approach that uses generative and discriminative machine learning models to more accurately estimate the symptom severity of patients and adjust therapy accordingly. A support vector machine is used as the representative algorithm for discriminative machine learning models, and the Gaussian mixture model is used for the generative models. Therapy is effected using the state estimates obtained from the machine learning models together with a fuzzy controller in a *critic-actor* control approach. Both machine learning model configurations achieve PD suppression to desired state in 7 out of 9 cases; most of which settle in under 2 s.

## Introduction

Continuous deep brain stimulation (DBS) for Parkinson's disease (PD) uses high frequency stimulation to ameliorate patient condition. However, this induces side effects in patients and shortens pacemaker battery life (Little et al., 2013). Both can be resolved using adaptive deep brain stimulation (aDBS). Adaptive DBS driven by feedback signals provides an approach that optimizes clinical benefits whilst minimizing side effects and battery depletion (Little et al., 2013; Arlotti et al., 2016). A commonly adopted feedback signal for aDBS are local field potentials (LFP) (Arlotti et al., 2018). LFP are used due to their correlation to patient clinical states and the ease with which they can be acquired (Priori et al., 2012; Little et al., 2016). In conventional DBS, programming of stimulation parameters are done by trained clinicians (Picillo et al., 2016). Thus, aDBS techniques that imitate human reasoning into decision making could be adopted—an example of which is fuzzy control.

**Figure 1:**
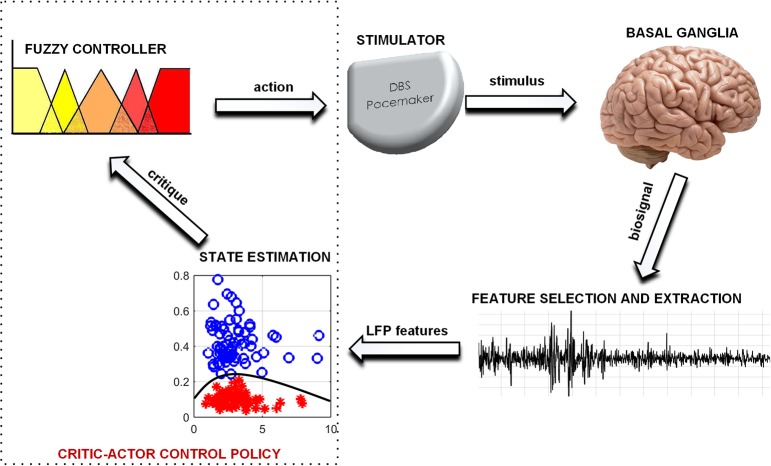
A typical scheme for adapting DBS using PD state estimates.

Fuzzy control is found in numerous applications for closed loop therapy (Zarkogianni et al., 2011; Soltesz et al., 2013; Zavitsanou et al., 2016). It has the potential to achieve a level of expertise close to (and possibly better than) human expertise in therapy modulation (Barro and Marin, 2002). However, the capabilities of fuzzy control are dependent on the level of sophistication of its rules and input signal. In this paper, state estimates are used as input to a fuzzy controller to achieve a *critic-actor* control policy as shown in Figure 1. It leverages on a machine learning model

as the *critic* and a fuzzy controller as the *actor*. This individualizes therapy by means of patient-specific state estimates which are obtained through the machine learning models. Machine learning models were selected because of their ability to create adaptable models for complex signals using statistical attributes from the signals (Sajda, 2006). The choice of fuzzy control was driven by their ability to provide computationally efficient and robust decision making. Consequently, as more knowledge on PD and DBS is gained, the fuzzy rules could be updated, which provides an adaptable control scheme. The scheme has the potential to be developed into a fully implantable closed-loop DBS system.

The rest of the paper is organized as follows. Section Machine Learning for Disease Tracking describes the methods adopted for disease tracking. Sections Models and Metrics and Fuzzy Controller Design describe the materials used for implementing the adopted methods. Section Performance Evaluation presents the results obtained. Sections Discussion and Conclusion present discussion and concluding remarks, respectively.

## Machine Learning for Disease Tracking

Disease tracking is important because dynamic changes in PD pathophysiology could help inform treatment strategies. This can be achieved using machine learning models as they provide insights on disease progression. In brain machine interface applications, machine learning provides the ability to notify caregivers of life-threatening events related to chronic disease diagnosis and management (Johnson et al., 2016; Mohammed and Demosthenous, 2018). Using closed-loop control strategies, this useful information can be used to generate actionable outputs—mostly from stimulation devices—to mitigate patient conditions (Csavoy et al., 2009). Machine learning models for disease tracking are intended to achieve one of two outcomes: prediction or state estimation. For optimal delivery of bio-electronic therapy, prediction is the most desirable outcome. Nevertheless, early and accurate state estimation can be used to adjust therapy to suit patients' needs. State estimation tracks fluctuations in PD symptom severity so that stimulation can be modulated correspondingly. Machine learning algorithms can be used to obtain state estimates. Generally, machine learning algorithms are classified into supervised (using labeled data), semi-supervised (using partly labeled data) and unsupervised (using unlabeled data) learning algorithms. This work will focus on the use of supervised learning algorithms.

### Supervised Learning Algorithms

These algorithms are not only concerned with detecting patient states, but can also be used in understanding the evolution of the pathophysiological processes in patients; thus, modeling transitions between various states in a disease. Supervised learning algorithms are divided into discriminative and generative machine learning models. For both algorithms, the major pre-processing approach adopted before state estimation is scaling the features using mean normalization. This is represented mathematically as follows,

(1) $$x_{j\_new}{}^{\left(i\right)}=\frac{x_{j}{}^{\left(i\right)}\;-\;\mu_{j}}{s_{j}}$$

where $\mu j=\frac{1}{m}\sum_{i=1}^{m}x_{j}{}^{\left(i\right)}$ is the mean of feature *j*, ${x_{j}}^{\left(i\right)}$ is feature *j* of training example *i* (with a total of *m* training examples ${x_{1}}^{\left(i\right)},{x_{2}}^{\left(i\right)},\ldots {x_{m}}^{\left(i\right)}$) and *s*_*j*_ is the standard deviation of feature *j*. Feature scaling using mean normalization scales features such that features have a comparable range of values.

Discriminative models focus on detecting disease or non-disease states, in this case PD and non- PD states. On the other hand, generative algorithms are particularly useful in applications were the sequence of transition between states is essential in determining future states, like in sleep-stage monitoring applications (Rossow et al., 2011). In aDBS, they can be principally useful in applications were stimulation parameters are defined by the evolution of the sensed neural potentials.

### Representative State Estimators

**Figure 2:**
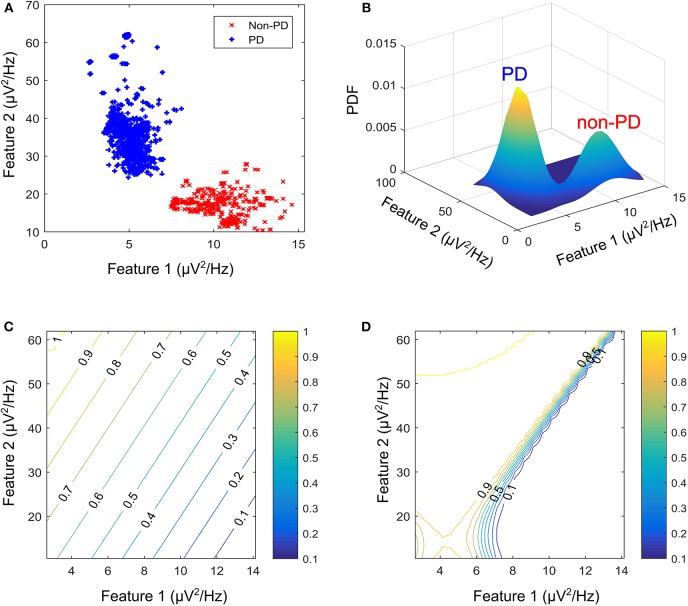
Contour plot for state estimates over a feature space for the machine learning models. **(A)** Example feature space showing PD and non-PD examples for dataset C. **(B)** Probability density function (PDF) for PD and non-PD training examples in **(A)**. **(C)** Contour plot for state estimates using SVM, with a range from 0 to 1 representing levels of severity from non-PD to PD for **(A)**. **(D)** Contour plot for state estimates using GMM, with a range from 0 to 1 representing levels of severity from non-PD to PD for **(A)**. The two features are, Feature 1 (21–26 Hz band) and Feature 2 (18–23 Hz band).

Generative algorithms model the data based on the joint probability distributions between its classes (PD and non-PD) while discriminative algorithm models data based on their conditional probability distribution. One example in each of the two models was used to test the soundness of the proposed framework for aDBS. A linear kernel support vector machine (SVM) was selected as the representative algorithm for the discriminative models while the Gaussian mixture model (GMM) was selected for the generative algorithms. SVM and GMM were selected because of their computational efficiency compared to other similar algorithms. Figure 2 shows the contour plot for features space using the conditional probability from the SVM as state estimate and the joint probability from the GMM as state estimate. PD regions are points on the plot where the probability is >0.5, while non-PD regions are those in which the probability is <0.5. Thus, from non-PD to severe PD is a transition in probability from 0 to 1.

The SVM uses the widest margin between differing states to discriminate. For a linear SVM kernel, the discriminating function *f*_SVM_(*x*), used in classifying test cases is obtained using the training examples as in Cristianini and Shawe-Taylor (2000).

(2) $$\begin{array}{r} f_{\text{S}\text{V}\text{M}}\left(x\right)=\sum_{\text{i}}{y_{i}\alpha }_{\text{i}}\left(x_{\text{i}},\;x\right)+b \end{array}$$

where *x*_*i*_ are the support vectors and their labels *y*_*i*_, *x* is the test case, (*x*_*i*_, *x*) is the kernel transformation (linear kernel), α_*i*_ is a weight vector and *b* represents the classification threshold. Figure 2C depicts the state estimates obtained using SVM on the feature space in Figure 2A, whose probability density function (PDF) is shown in Figure 2B.

The GMM estimates conditional probability using a weighted sum of a number of PDFs. These PDFs are used to form the Gaussian models. The weighted Gaussian functions f_GMM_(x) modeling the underlying processes are

(3) $$f_{GMM}\left(x\right)\;=\sum_{i\;=\;1}^{N}w_{i}\;exp(-{\left(\vec{x}\;-\;\mu_{i}\right)}^{T}\Lambda_{i}(\vec{x}\;-\;\mu_{i}))$$

where *w*_*i*_ is the weight assigned to a particular Gaussian model *i*, $\vec{x}$ is the input feature vector, μ_*i*_ is the mean vector and Λ_*i*_ is the covariance matrix. The major assumption employed in GMM is that the population of feature vectors can be represented by *N* Gaussian models. Thus, two Gaussian models are fitted in the training data, in order to represent each of the patient states (PD and non-PD). Figure 2C shows the state estimates obtained using SVM on the feature space in Figure 2A, whose PDF is shown in Figure 2B.

## Models and Metrics

The proposed model in Figure 1 consists of a basal ganglia network, a feature extraction stage, a state estimation stage for diagnosing PD severity and an adaptive stimulator for delivering therapy. The basal ganglia network uses LFP recordings to mimic the underlying mechanism of PD. LFP signals from the basal ganglia model are applied to a feature processing stage, and the output from this stage is applied to a state estimation stage. Stimulation parameters are adjusted based on patient state estimates. The model was developed using custom SIMULINK blocks. The SIMULINK blocks were implemented using level-2 MATLAB S-functions. This was used to validate the complete aDBS system.

### Basal-Ganglia Network Model

**Figure 3:**
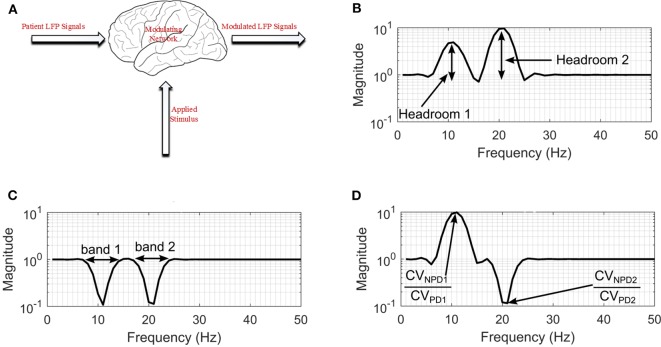
Modulating network used to simulate the effect of DBS on neuronal signals. **(A)** Basal-ganglia network model. **(B)** Frequency response for configuration with non-PD having higher amplitude in both bands. **(C)** Frequency response for a configuration with PD having higher amplitude in both bands. **(D)** Frequency response for a configuration with non-PD having higher amplitude in band 1, and PD having higher amplitudes in band 2. *CV*_*NPD*_ is the coefficient of variation for non-PD LFP signal and *CV*_*PD*_ is the coefficient of variation for PD LFP signal.

In order to validate these methods a basal ganglia model using LFP recordings obtained from measurements made on patients exhibiting a combination of bradykinesia and/or rigidity during the onset of PD, with less noticeable tremor was employed. The network which is shown in Figure 3A, consists of: patient LFP signals, modulating network and the modulated LFP signal.

#### Patient LFP Signals

These are LFP signals consisting of PD and non-PD periods synthesized from real-life LFP recordings. The LFP synthesis, involved fitting autoregressive moving average (ARMA) models to the LFP recordings to produce semi-synthetic LFP signals. Fitting an ARMA model provides the flexibility to manipulate the signal characteristics such that all underlying conditions can be represented. Also, it offers the opportunity to generate LFP signals consisting of PD and non-PD episodes of variable duration. The LFP dataset consists of LFP recording for nine patients. The recordings were obtained from the subthalamic nucleus (STN) of subjects exhibiting a combination of bradykinesia and/or rigidity during the onset of PD, with less noticeable tremor.

**Figure 4:**
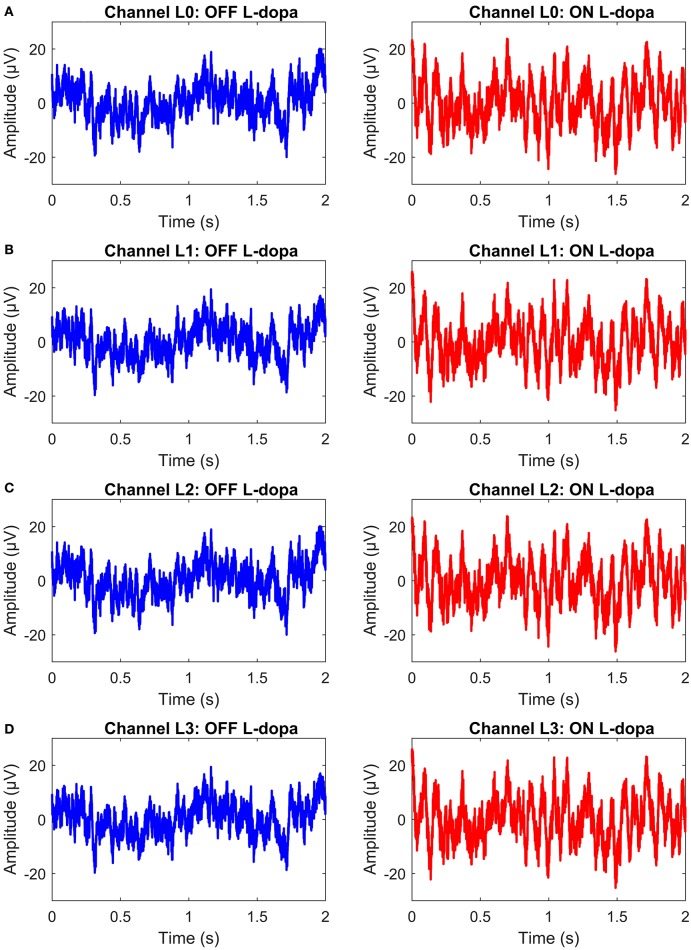
A snapshot of OFF and ON L-dopa recordings (representing PD and non-PD LFP recordings) of the left DBS lead of dataset A. **(A)** OFF and ON L-dopa recordings of electrode L0. **(B)** OFF and ON L-dopa recordings of electrode L1. **(C)** OFF and ON L-dopa recordings of electrode L2. **(D)** OFF and ON L-dopa recordings of electrode L3.

The permanent quadri-polar macro-electrode used was model 3,389 (Medtronic Neurologic Division, Minneapolis, MN) consisting of 4 platinum-iridium cylindrical contacts. Its contacts were numbered 0, 1, 2, and 3, with 0 being the most caudal and 3 being the most cranial for both right and left electrodes—making a total of eight monopolar channels for each patient. The recorded signals were amplified using a low-noise amplifier and band-pass filtered. Figure 4 shows a snapshot of OFF and ON L-dopa recordings of the left DBS lead for patient/dataset A. The complete LFP data synthesis process and a detailed description of the LFP recordings are provided in Mohammed et al. (2017). For the basal-ganglia model in Figure 3A, the applied stimulus regulates the patient LFP signal such that the modulated LFP characteristics are restored to those resembling non-PD LFP. Stimulation is not applied on detecting patient LFP with non-PD characteristics.

#### Modulating Network and Modulated LFP Signals

The therapeutic mechanisms of DBS on neuronal activities is still not clear. Various studies suggest that it reduces neuronal activities (Kiss et al., 2002), while others claim that it increases neuronal activities (Carlson et al., 2010). Later studies provide other alternative explanations (Chiken and Nambu, 2016). Generally, studies show that the frequency settings of DBS of the STN influence the motor symptoms of PD. For example, the study in Su et al. (2018), observed that frequency-specific effects can ultimately inform the frequency programming of STN-DBS in the clinical use. From the studies, what is clear is that DBS has a multimodal and modulating effect on neuronal activities at the stimulation site (Hell et al., 2019). In addition, the various clinical aspects related to bradykinesia and other PD symptoms are still unclear (Bologna et al., 2020). As such, to model the effect of stimulation on patient LFP signals, a black-box approach was used as shown in Figure 3A.

For the black box model, changes in the coefficient of variation (CV) of neuronal signals during DBS supports the hypothesis that modulating LFP signals is one of the mechanisms that can lead to PD suppression (Birdno and Grill, 2008; Dorval et al., 2010). PD symptoms have been found to correlate with beta band LFPs (Little et al., 2012, 2013; Grant and Lowery, 2013); gamma (Brown and Williams, 2005; Little and Brown, 2012; Brittain and Brown, 2014); and tremor (Heida et al., 2013) bands. Hence, the neuromodulatory effect of DBS on PD occurs in multiple bands. This prompted the two-degrees-of-freedom (2-DOF) changes in CV applied by the modulating network as shown in Figures 3B–D. For 2-DOF modulation in CV, the modulating network varies the amplitude of patient LFP signals in the two bands with the most pronounced variation between non-PD and PD bands as shown in Figure 3C. For both bands, the headroom of variation for the magnitude of the filter response is between 1 and the ratio of CV for non-PD to PD (CV_NPD_/CV_PD_), as is shown in Figure 3D. Figures 3B–D show all the cases of CV ratio between PD and non-PD for 2-DOF variation. Figure 3B shows a situation where CV_NPD_ in both bands is greater than CV_PD_. Figure 3C shows a situation where CV_NPD_ in both bands is less than CV_PD_. Finally, Figure 3D shows a situation where CV_NPD_ in one of the bands is greater than CV_PD_. This makes the modulating network unique for each patient since the frequency response of the modulating network is dependent on the relationship between PD and non-PD periods of each patient.

The modulated LFP signals are extracellular/LFP signals resulting from the modification of patient LFP signals by the modulating network. The modulated LFP signals are the signals monitored by aDBS in order to adjust the stimulation.

### Feature Extraction and Selection

For feature extraction, the fast Fourier Transform (FFT) is used to obtain time-frequency data. This is achieved by dividing the signal into windows and applying FFT to each window (Prandoni and Vetterli, 2008). Mathematically STFT is given by

(4) $$\begin{array}{r} X_{n}\;\left[m;k\right]=\;\overset{L-1}{\underset{n=\;0}{\sum }}x\left[m+n\right]\;e^{-j\frac{2\pi }{L}nk} \end{array}$$

where *m* is the discrete time index, *L* is the window length into which the signal is split and *k* is the discrete frequency index. For this application, the time-stamped measurements are split into 2 s overlapping epochs, with 50% overlap between epochs. In addition, the power bands (features) are divided into 5 Hz bands, with 3 Hz overlap between bands; 0 to 5, 3 to 8 Hz, … 45 to 50 Hz. This provides a total of 16 features. The window is chosen such that a balance between time and frequency resolution is obtained.

More so, feature selection involves reducing the number of features that will be used for state estimation. For this study, the maximum ratio method is used (Mohammed et al., 2017). It starts by identifying the channel having the two bands with the most pronounced variation in activity between PD and non-PD LFP signals. The goal is to obtain the frequency bands that make state estimation easier and computationally efficient. The maximum ratio method is a computationally simple method. A more detailed description is presented in Mohammed et al. (2017).

### Stimulation Parameters

Stimulation is used to respond to fluctuations in the dynamics of patient LFP data. The estimated patient state is applied to the fuzzy controller and the fuzzy controller determines the appropriate stimulation parameters. The fuzzy controller is designed in section Fuzzy Controller Design. The poorly understood mechanisms for DBS makes the selection of stimulation parameters (i.e., amplitude, frequency and pulse duration) difficult (Kuncel and Grill, 2004). Experimental studies have been undertaken regarding the most beneficial stimulation parameter for reduction in motor symptoms. Some studies suggest that there are more noticeable improvements when stimulation frequency is adjusted (Moreau et al., 2008; Xie et al., 2012; Belasen et al., 2016). However, other studies maintain that stimulation amplitude is more critical (Moro et al., 2002; Eusebio et al., 2011; Whitmer et al., 2012). More research has focused on stimulation frequency alone (Birdno and Grill, 2008; Baker et al., 2011; Brocker et al., 2013). Varying the stimulation frequency is essential for the therapeutic effects of STN-DBS on motor symptoms in PD (Su et al., 2018). Nevertheless, the major considerations in selecting stimulation parameters are patient responses to stimulation patterns and power consumption to conserve battery life (Kuncel and Grill, 2004). The consensus is that the most beneficial stimulation frequency occurs at 130 Hz (Birdno and Grill, 2008; Moreau et al., 2008; Vercruysse et al., 2014).

**Figure 5:**
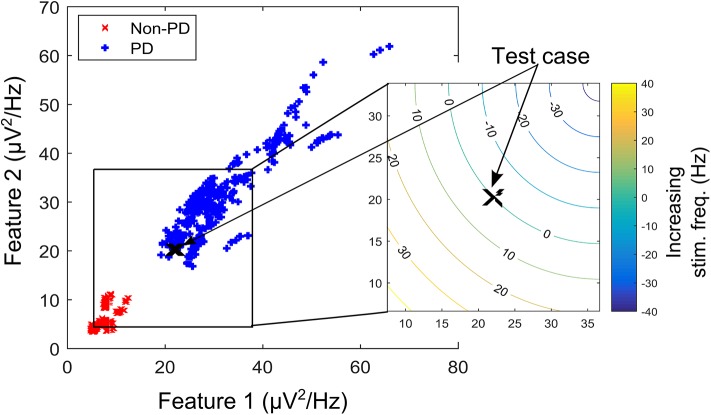
A contour plot depicting the effect of increasing/decreasing stimulation frequency on the transition path of a test case (in the XY-location marked “X”) over the feature space. Feature space is that of dataset B.

Based on the therapeutic benefits of varying the stimulation frequency, the fuzzy rules are designed to adjust the stimulation frequency. Adjusting the stimulation frequency modifies the modulating effect in a linear fashion as depicted in Figure 5. The headroom for the frequency response of the modulating network in Figure 3 (i.e., a magnitude response of between 1 and CV_NPD_/CV_PD_) corresponds to a stimulation frequency ranging from 0 to 90 Hz. This is shown in the contour plot of Figure 5, where increasing the stimulation frequency by 45 Hz moves the test case from the point marked X to the center of the non-PD cluster, while a decrease of 45 Hz moves it to the center of the PD cluster. In theory, a 90 Hz increase/decrease in stimulation frequency maintained over 2 s can move a test case from the center of one cluster to the other (PD to non-PD cluster or vice versa). The range of stimulation frequency is between 0 and 180 Hz, which is within the limit for conventional DBS.

### Evaluation Metrics

In assessing the performance of the different state estimator-based approaches, three measures that are indicative of accuracy, latency and computational complexity have been used.

#### Accuracy

The state estimators are evaluated using Mathews correlation coefficient (MCC) and weighted classification error (WCE). MCC and WCE are balanced measures used in assessing state estimator quality that can be used even for cases with skewed classes. MCC measures the correlation coefficient between the observed and predicted binary classifications. It has a range between −1 (total disagreement) and +1 (total agreement); with 0 representing a random prediction. Mathematically,

(5) $$\begin{array}{r} \text{M}\text{C}\text{C}\;=\;\frac{\left(TP\times TN\right)-\left(FP\times FN\right)}{\sqrt{\left(TP+FN\right)\;\left(TP+FP\right)\;\left(TN+FP\right)\;\left(TN+FN\right)}} \end{array}$$

where TP are the true positives, FP the false positives, FN the false negatives and TN the true negatives. The major shortcoming of MCC is that it can only be used when one of the denominators TP + FN, TP + FP, TN + FP and TN + FN is not a zero. For WCE, it can be represented mathematically as,

(6) $$\begin{array}{r} \text{W}\text{C}\text{E}\;=\;\frac{1}{2}\left(\frac{FP}{FP\;+\;TN}+\;\frac{FN}{TP\;+\;FN}\right). \end{array}$$

The first part of Equation (5) represents type I error (false-positive rate), while the second part represents type II error (false-negative rate). In Equation (5), WCE uses equal weights to compute the average of type I and type II error.

#### Latency

Detection latency in this work, measures the total time required by the system (or controller) to settle to the modal state interval for non-PD defined by the fuzzy controller. For SVM driven control policy, the modal state interval is between 0.15 and 0.35, while for GMM, it is a state between 1 × 10^−8^ and 0.1. This were empirically obtained considering that from non-PD to severe PD is a transition from 0 to 1.

#### Computational Complexity

In this work, the primary concern is the computational cost of the critic-actor control algorithm consisting of the state estimator and the fuzzy controller. Computational cost consists of two components, number of operations (NOP) and memory requirements. NOP is measured using the number of additions and number of multiplications. It can be represented mathematically as,

(7) $$NOP=N_{add\left(sub\right)}+Res\times N_{mult\left(div\right)}$$

where *N*_add(sub)_ is the number of 1-bit additions or subtractions; *N*_mult(div)_ is the number of 1-bit multiplications and divisions; and Res is the resolution of the data converter used. For memory estimates, the number of 1-bit registers required are obtained.

## Fuzzy Controller Design

Based on parkinsonian state estimates, fuzzy rules are defined to regulate stimulation. The fuzzy controller modifies the stimulation parameters applied to the modulating network to suppress PD-related oscillations. A fuzzy controller was chosen because it uses a reasoning which is similar to human reasoning and decision making. This makes it superior in handling non-linearity and uncertainty compared to schemes like proportional-integral-derivative controllers, lead-lag and state feedback control (Feng, 2006; Wu et al., 2017). Fuzzy controller design essentially involves the following:

- Choosing the fuzzy controller inputs and outputs.
- Choosing the pre-processing that is required for the controller inputs and the post-processing for the controller outputs.
- Designing the four components of the fuzzy controller (rule-table, inference mechanism, fuzzification and defuzzification).

**Figure 6:**
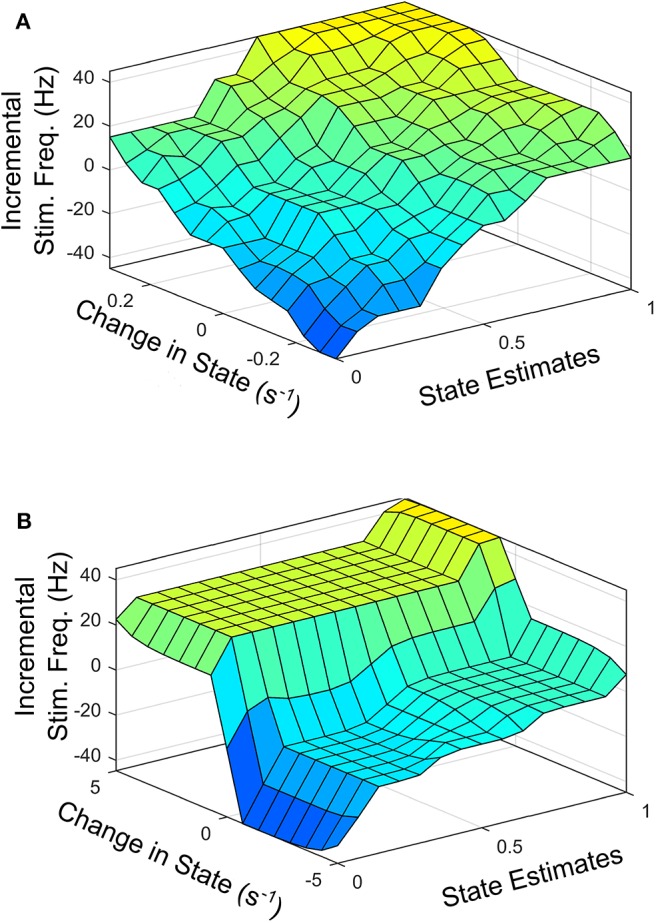
Surface plot for input-output relationship for: **(A)** SVM based controller, **(B)** GMM based controller.

To facilitate the design of the fuzzy controller, Figures 6A,B shows the desired average profile for the effect of incremental stimulation frequency for all possible input combinations for the SVM and GMM driven approaches, respectively.

Figure 6 represents the average 3-D profile that maps inputs (state estimates and change in state) to outputs (stimulation frequency). To obtain the profile or each patient dataset, training examples at discrete points on the feature space representing states estimates ranging from 0 to 1 in steps of 0.1 are identified. For training examples at each discrete point on the feature space, stimulation frequency is increased in steps from −*45 to* +*45* Hz (in steps of 5 Hz). The corresponding rate of change in patient state is obtained for each discrete pair consisting of patient state estimate and applied stimulation frequency. This produces a mapping of three variables (patient state estimates, change in state and applied stimulation frequency). This means, for every patient state, there is an applied stimulation frequency that results in a specific rate of change in patient state. The process is repeated for all nine patient datasets and the average for the various profiles are obtained as Figures 6A,B. For the SVM based approach, Figure 6A represents the average 3-D profile that maps state estimates and change in state to stimulation frequency. This is represented by Figure 6B for the GMM based approach. The average profiles in Figure 6 are used to guide the rule-tables for controlling PD suppression.

The profile for the change in state (measured in s^−1^) targets a settling time of between 1 and 1.5 s from the center of the modal class of the PD state (with a probability 0.75 for SVM, and 0.9999 for GMM) to the center of the modal class of the non-PD state (with a probability 0.25 for SVM and 1 × 10^−4^ for GMM). From Figure 6 it is obvious that from a PD state of 1, the SVM-driven approach has a more gradual descent, while the GMM has a sharper descent at the edges, plateaus for a range of input values in which change in input only causes a slight change in stimulation frequency before it finally descends steeply. This surface plot guided the choice of membership function and rule table for the fuzzy controller, which are normally chosen heuristically. The input-output relationship was obtained using the average profile for state estimate and incremental stimulation frequency which are depicted in Figures 2, 5, respectively.

### Fuzzification

This is the encoding step. It modifies the inputs so that they can be interpreted and compared to the rules in the rule-table. The controller inputs are converted to information usable by the inference mechanism. Obtaining a value for an input variable and finding the numeric values of the membership functions that are defined for that variable. It can also be seen as an encoding of the fuzzy controller inputs. The encoded information is used in the fuzzy inference process that begins with matching.

### Fuzzy Rules and Membership Functions

**Figure 7:**
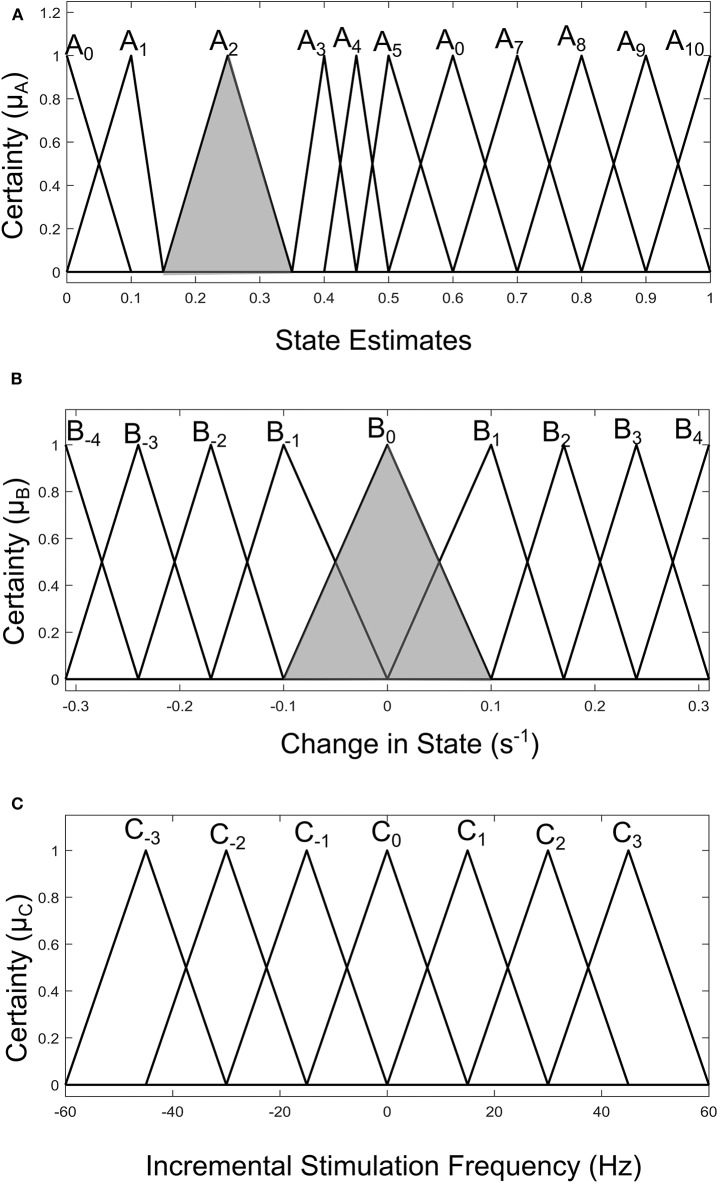
Input-output membership functions for the fuzzy controller driven by SVM state estimates. **(A)** Membership functions for the state estimates. **(B)** Membership functions for the rate of change in state. **(C)** Membership function for the incremental stimulation frequency.

Fuzzy rules and membership functions are obtained by studying the plant dynamics (using modeling and simulation), based on these, a set of control rules that make sense are adopted. This makes fuzzy controller design subjective and dependent on expert designer (Passino and Yurkovich, 1998). In addition, the adaptable nature of a fuzzy controller makes a suitable candidate, since the mechanisms of DBS are still under debate. The control scheme uses a two-input one-output fuzzy controller. The inputs are PD state estimate and the rate of change in state. The state estimates and rate of change in state quantify the dynamics of the underlying process to enable control. State estimates are obtained using SVM and GMM. The output is the incremental stimulation frequency. Based on the contour plot of the state estimates in Figures 2C,D, and the contour plot depicting the effect of stimulation frequency in Figure 5, triangular membership functions were used for the inputs and output of the SVM driven approach. While for the GMM based approach, Gaussian functions were adopted. The rule table for the SVM-driven approach is shown in Table 1. It is obtained using the 3-D profile in Figure 7A representing the mapping between inputs (state estimates and change in state) and outputs (stimulation frequency). The input membership function for the rules in Table 1 are summarized in Figures 7A,B. While the membership functions for the output (incremental stimulation frequency) is summarized in Figure 7C.

**Table 1:** Rule table for control policy using SVM for state estimation.

**Incremental stimulation frequency**		**Change in state**								
		**B_**−4**_**	**B_**−3**_**	**B_**−2**_**	**B_**−1**_**	**B_**0**_**	**B_**1**_**	**B_**2**_**	**B_**3**_**	**B_**4**_**
State	A_0_	C_−3_	C_−3_	C_−2_	C_−2_	C_−1_	C_−1_	C_−0_	C_−0_	C_1_
	A_1_	C_−2_	C_−2_	C_−1_	C_−1_	C_−1_	C_−0_	C_0_	C_1_	C_1_
	A_2_	C_−2_	C_−1_	C_−1_	C_−0_	C_−0_	C_−0_	C_1_	C_1_	C_1_
	A_3_	C_−1_	C_−1_	C_0_	C_0_	C_0_	C_1_	C_1_	C_2_	C_2_
	A_4_	C_0_	C_0_	C_0_	C_0_	C_1_	C_1_	C_2_	C_2_	C_2_
	A_5_	C_0_	C_0_	C_0_	C_1_	C_1_	C_2_	C_2_	C_3_	C_3_
	A_6_	C_0_	C_1_	C_1_	C_1_	C_2_	C_2_	C_3_	C_3_	C_3_
	A_7_	C_1_	C_1_	C_1_	C_1_	C_2_	C_2_	C_2_	C_3_	C_3_
	A_8_	C_1_	C_1_	C_1_	C_2_	C_2_	C_2_	C_3_	C_3_	C_3_
	A_9_	C_1_	C_1_	C_2_	C_2_	C_2_	C_3_	C_3_	C_3_	C_3_
	A_10_	C_1_	C_2_	C_2_	C_2_	C_2_	C_3_	C_3_	C_3_	C_3_

**Figure 8:**
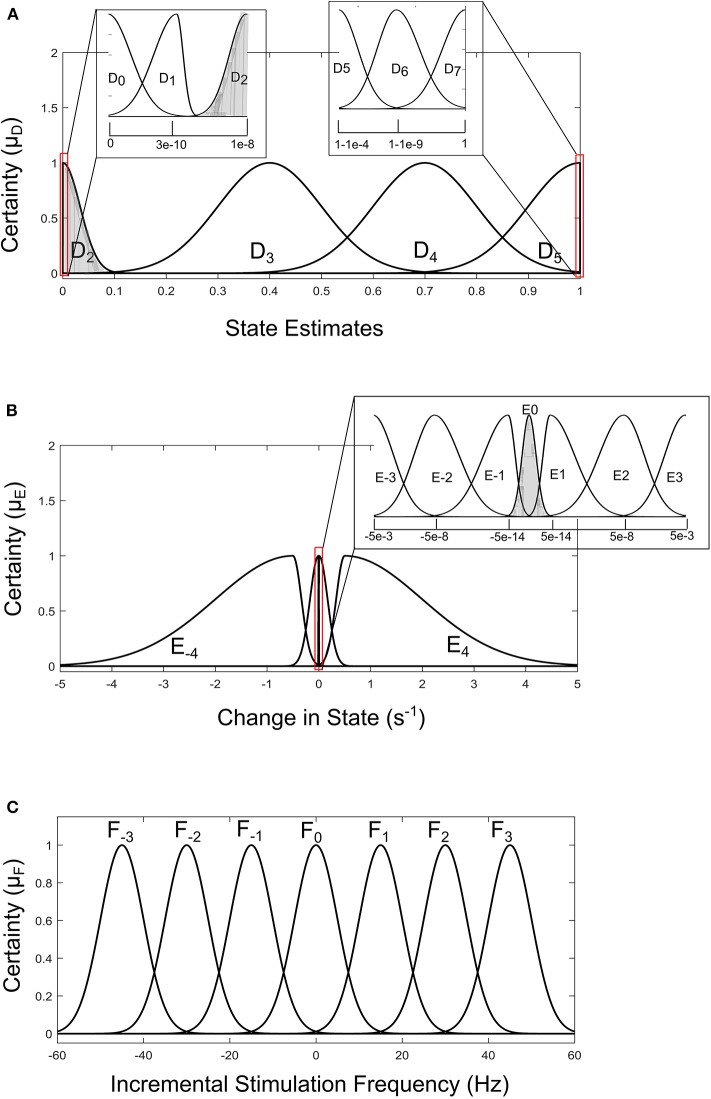
Input-output membership functions for the fuzzy controller driven by GMM state estimates. **(A)** Membership functions for the state estimates. **(B)** Membership functions for the rate of change in state. **(C)** Membership function for the incremental stimulation frequency.

For the GMM-based control approach, its rule table is shown in Table 2. It uses Gaussian membership functions. Its input membership functions for the rules in Table 2 are summarized in Figures 8A,B. While the membership functions for the output (incremental stimulation frequency) is shown in Figure 8C. The universe of discourse for the state estimates is [0, 1] as can be seen in Figure 7A and Figure 8A for the SVM and GMM, respectively. The input fuzzy sets for the SVM are represented by alphanumeric variables A_0_ A_1_ … A_10_, and that of the GMM is D_0_, D_1_ … D_7_. This means for state estimates, the SVM driven approach has eleven fuzzy sets and the GMM driven approach has eight fuzzy sets. The membership functions for the SVM and GMM driven controllers are summarized in Figures 7, 8, respectively.

**Table 2:** Rule table for control policy using GMM for state estimation.

**Incremental stimulation frequency**		**Change in state**								
		**E_**−4**_**	**E_**−3**_**	**E_**−2**_**	**E_**−1**_**	**E_**0**_**	**E_**1**_**	**E_**2**_**	**E_**3**_**	**E_**4**_**
State	D_0_	E_−3_	E_−3_	E_−2_	E_−1_	E_−1_	E_−1_	E_−0_	E_−0_	E_1_
	D_1_	E_−2_	E_−2_	E_−1_	E_−1_	E_−1_	E_−0_	E_0_	E_1_	E_1_
	D_2_	E_−2_	E_−1_	E_−1_	E_−0_	E_−0_	E_−0_	E_1_	E_1_	E_2_
	D_3_	E_−1_	E_−1_	E_0_	E_0_	E_0_	E_1_	E_1_	E_2_	E_2_
	D_4_	E_0_	E_0_	E_0_	E_1_	E_1_	E_1_	E_2_	E_2_	E_2_
	D_5_	E_0_	E_0_	E_1_	E_1_	E_2_	E_2_	E_2_	E_2_	E_3_
	D_6_	E_1_	E_1_	E_1_	E_2_	E_2_	E_2_	E_3_	E_3_	E_3_
	D_7_	E_1_	E_1_	E_2_	E_2_	E_2_	E_3_	E_3_	E_3_	E_3_

For the second input which is change in state, the fuzzy sets of the SVM driven approach are represented by alphanumeric variables B_−4_ … B_0_ … B_4_, making a total of nine fuzzy sets. Their membership functions are summarized in Figure 7. From Figure 7, it can be seen that negative subscripts represent a change from one toward zero (PD to non-PD) and positive subscripts represent a change from zero toward one (non-PD to PD). This is the same for the change in state of the GMM-driven approach with fuzzy sets represented by alphanumeric variables E_−4_ … E_0_ … E_4_, and their respective membership functions summarized in Figure 8. As summarized in Figures 7, 8, the universe of discourse for the SVM-driven approach is [−0.31, 0.31] s^−1^ and that of the GMM-driven approach is [−5, 5] s^−1^. The fuzzy sets representing the output (incremental stimulation frequency) are labeled C_−3_ … C_3_, for the SVM approach and that of the GMM are labeled F_−3_ … F_3_. Like in the fuzzy sets for the change in state, the negative subscripts represent an output representing a reduction in stimulation frequency, while a positive subscript represents an output resulting in an increase in stimulation frequency. Both have a universe of discourse of [−60, 60] Hz. Based on heuristics, the SVM rule-table has an 11 × 9 array making a total of 99 possible rules, which are summarized in Table 1. For the GMM rule-table in Table 2, it is made up of an 8 × 9 array making a total of 72 possible rules.

The desired fuzzy set for the SVM driven approach is shaded in Figure 7. The desired fuzzy set for state estimates is between the intervals 0.15 and 0.35 (represented by A_2_ in Figure 7A). This represents the modal class for non-PD cases. In terms of the change in state, the desired interval is between −0.1 s^−1^ and 0.1 s^−1^ (represented by B_0_ in Figure 7B). The modal class interval for the state estimate (A_2_) was made not to overlap with other classes to avoid ambiguity in fuzzy quantification. The outermost membership functions for the inputs can be seen to saturate and values outside the range are grouped to their closest fuzzy set. However, this is not the case for the output, due to the requirement for a defined output value at any instant in time. For the GMM driven approach, the desired input values are: 1 × 10^−8^−0.1 for state estimates (represented by D_2_ in Figure 8A) and −5 × 10^−14^ s^−1^ to 5 × 10^−14^ s^−1^ for change in state (represented by E_0_ in Figure 8B). Fuzzy rules and definition of membership function are subjective and are dependent on the expert designer. That is why a wide desired range was selected in both approaches to ensure convergence. In addition, the selected range represents the modal state for stable and non-disease conditions when projected to the patient feature space, which could be demonstrative of symptom severity. The membership functions and fuzzy rules were defined carefully based on the gradation of the state estimates on the patients feature space. This was to enable a gradual and deliberate PD suppression as against abrupt and jerky response.

### Inference Mechanism

The inference mechanism generally involves two steps: premise quantification and determining conclusions. Premise quantification compares the premise of all rules to the controller inputs to determine which rules are applicable to the current situation. It involves determining the certainty with which rules apply. The recommendations from rules that we are more certain with are adopted. Next is the determination of conclusions. This decides the control action to take using the applicable rules at the current time instant. The conclusions are characterized with a fuzzy set that represents the certainty with which the input should take various values. Premise quantification using the minimum f the applicable rules is adopted, while conclusion determination is obtained by ANDing the applicable rules.

### Defuzzification

This is the final operation of the fuzzy controller. It operates on implied fuzzy sets (output fuzzy sets) produced by the inference mechanism. It combines the effects of the various fuzzy sets to produce the “most certain” controller output (plant output). Defuzzification can be considered as decoding. As the fuzzy sets produced by the inferencing process (implied fuzzy sets) is converted to numerical controller outputs. The center of gravity (COG) method for combining recommendations was adopted. More detail of defuzzication is given in Passino and Yurkovich (1998). From both Tables 1, 2, the pattern of rule consequents shows a certain symmetry. For states estimates approaching a state of 1 and having a positive rate of change in state (positive subscript i.e., moving from non-PD to PD), there is a positive increase in stimulation frequency (positive subscript). Similarly, for state estimates approaching 0 and having a negative rate of change in state (negative subscript i.e., moving from PD to non-PD), the incremental stimulation frequency is negative (negative subscript). Note, the diagonals of near zero for the incremental stimulation frequency from state A_0_ to state A_6_, for the SVM and A_0_ to state A_5_, for the GMM in Tables 1, 2, respectively.

## Performance Evaluation

### PD Suppression

**Figure 9:**
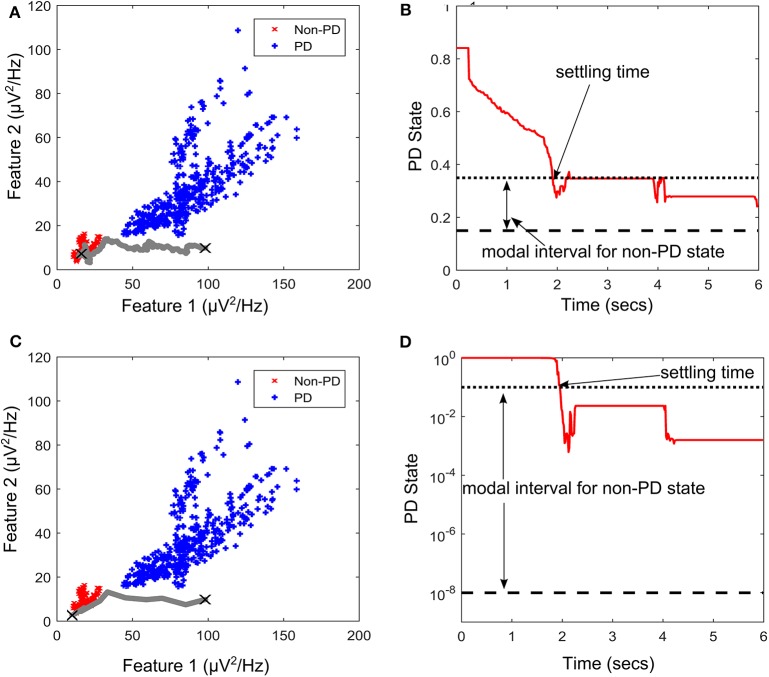
State transition of PD suppression on feature space of patient/dataset E. **(A)** Showing PD state transition on a feature space using SVM for state estimation, with “X” markers showing start (from PD) and settling (non-PD) positions. The feature space trajectory is indicated in gray. **(B)** PD state profile for PD suppression using SVM to obtain state estimates. It depicts the modal interval for the non-PD state when SVM is used for state estimation. **(C)** Showing PD state transition on a feature space using GMM for state estimation, with “X” markers showing start (from PD) and settling (non-PD) positions. The feature space trajectory is indicated in gray. **(D)** PD state profile for PD suppression using GMM to obtain state estimates. It depicts the modal interval for the non-PD state when GMM is used for state estimation.

PD suppression is depicted in Figure 9 using the GMM and SVM driven approaches. From Figure 9A which depicts SVM state estimation, it can be seen that the test case travels from the PD region and converges at the non-PD region as desired. It is also the case for the GMM approach in Figure 7C, but with a smoother trajectory. Figures 9A,C show the feature space profile and Figures 9B,D display the time profile. For the time profile, it can be seen that both cases cross the desired interval exactly after 2 s and both present the same settling profile. After settling, the SVM based approach has a mean PD state of 0.3137 and GMM-driven approach has a PD state of 1.3 × 10^−2^, both of which fall within the desired range.

**Figure 10:**
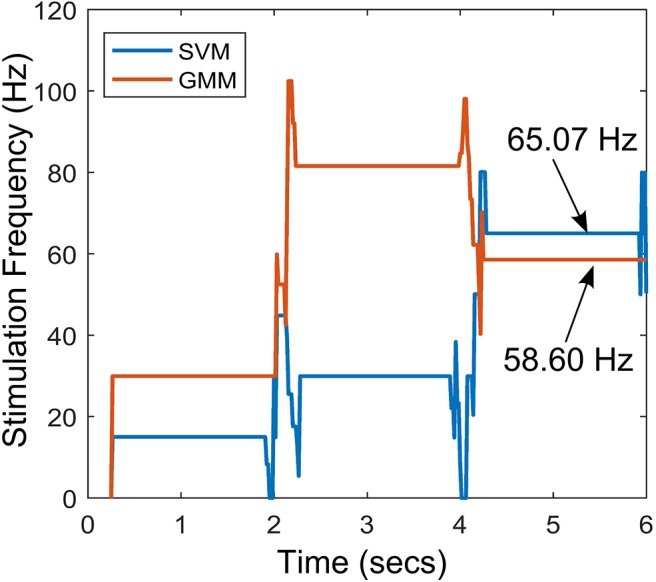
Stimulation profile for the state transition shown in Figure 9.

The stimulation profile for both cases is shown in Figure 10. Both cases present almost the same stepwise pattern, with the SVM having a more gradual ascent to the required stimulation frequency compared to the GMM which overshoots before finally settling. The settling stimulation frequency for both cases are not far apart. The feature space profile on the feature space and the time profile (both in Figure 9) display a stable PD suppression profile. In addition, the stimulation profile in Figure 10 also displays a stable stimulation profile. Both of these are indicative of a stable PD suppression.

For the rest of the datasets, Table 3 summarize their mean PD state and settling time. For the mean PD state in Table 3, the SVM has a lower quartile of 0.2514 and an upper quartile of 0.316, which both fall within the desired range (0.15–0.35). For the GMM, it has an upper quartile of 0.085 and a lower quartile of 2.5 × 10^−4^, which are both within the desired range (1 × 10^−8^ −0.1). For the settling times in Table 3, the SVM-driven approach has a median of 1.5 s, lower quartile of 1.25 s and an upper quartile of 1.875 s. While for the GMM, it has a median of 1.25 s, lower quartile of 0.25 and an upper quartile of 1.75 s. This shows that on average, the GMM based approach settles faster than the SVM based approach; however, the GMM has more variation in settling time as shown in Table 3.

**Table 3:** Average settling time and settling state for various patient datasets.

**Datasets**	**Average settling time (s)**		**Average settling state**	
	**SVM**	**GMM**	**SVM**	**GMM**
A	1.25	0.50	0.3237	0.0034
B	1.50	1.75	0.2584	0.1640
C	1.50	1.25	0.2802	3.5 × 10^−4^
D	1.25	0.25	0.2547	4.5 × 10^−9^
E	1.75	1.75	0.3137	0.0130
F	1.75	1.75	0.2542	0.0720
G	2.25	2.25	0.4950	0.1245
H	2.25	0.25	0.1735	4.4 × 10^−20^
I	0.50	0.25	0.2431	0.0042

### Performance of State Estimators

To assess the quality of the SVM and GMM state estimators, the MCC and WCE which are skew insensitive measures were used. The MCC measured the correlation coefficient between the original dataset and the models fitted using each of the state estimators. On the other hand, the WCE consisted of weightings of type I and type II error. This was because in aDBS, high false positive-rate will result in administering stimulation when it is not required, and this may lead to stimulation induced side effects (Baizabal-Carvallo and Jankovic, 2016). High false-negative rate will result in the non-administering of stimulation when it may be required, which could worsen patient condition (Hacker et al., 2015). The real-time detection performance of the state estimator was investigated. Both models used 128 training examples and PD events were detected from 2 s overlapping epochs (with 50% overlap). Table 4 summarizes the average result obtained for each dataset for 100 Monte Carlo runs using 256 test cases (256 LFP epochs). For each of the nine test cases, there is a training (and hold-out/cross validation) phase then a test phase to validate the closed-loop architecture.

**Table 4:** State estimation performance of SVM and GMM on various patient data.

**Datasets**	**MCC**		**WCE**	
	**SVM**	**GMM**	**SVM**	**GMM**
A	0.3534	0.5273	0.3447	0.2204
B	1	0.8863	0	0.0771
C	1	1	0	0
D	1	0.9976	0	0.0016
E	1	1	0	0
F	0.9433	0.9433	0.0198	0.0198
G	0.4479	0.2347	0.3273	0.3757
H	1	0.9963	0	0.0012
I	0.7371	0.4343	0.1210	0.2943

For MCC in Table 4, both state estimators present a positive correlation for all datasets, with the SVM having a median of 1 and the GMM with a median of 0.9433. Of the 9 cases, both SVM and GMM have 7 cases with strong positive correlation (MCC ≥ 0.5). Only the state estimates of dataset G have a weak positive correlation in both cases. This is due to the high overlap between its PD and non-PD clusters which makes it difficult to fit the classifier to the data. From the MCC results, it can be seen that SVM fits the data better than the GMM. Similarly, the WCE results present a superior performance of the SVM over the GMM. The SVM presents a mean and median WCE of 9.03 and 0%, respectively. While the GMM presents a mean and median of 11 and 1.98%, respectively. This further confirms the superiority of the SVM over the GMM in fitting the data.

### Relative Complexity

**Figure 11:**
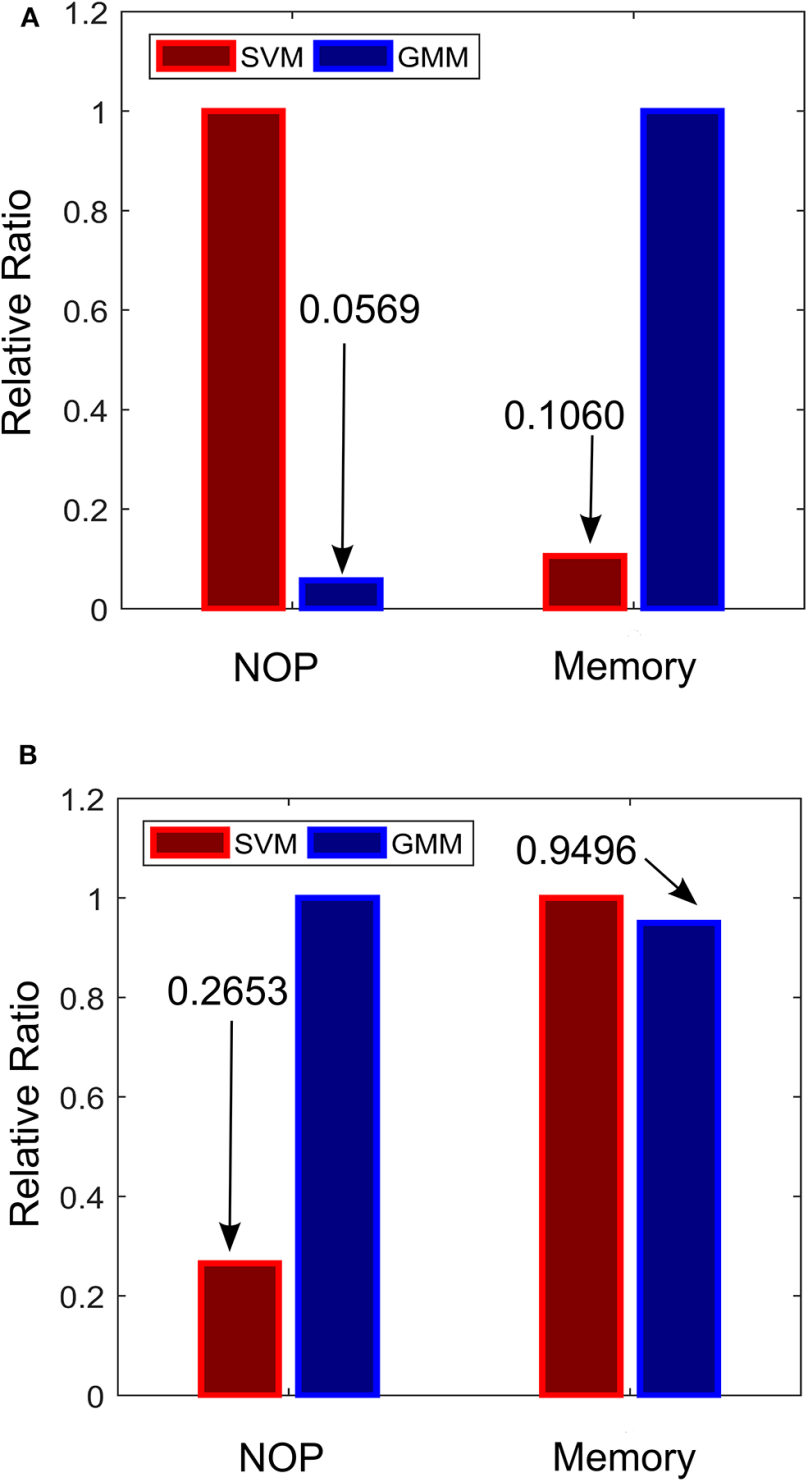
Relative complexity of the critic-actor control driven by GMM and SVM. **(A)** Normalized complexity for the state estimation stage. **(B)** Normalized complexity for the fuzzy control stage. Normalized to the maximum for all cases (*maximum* = *1*).

To ensure that the approach is effective for real LFP recordings, the semi-synthetic LFP were made from real LFP recordings to mimic PD progression in real LFP recordings. In addition, state estimators that are size and power conscious were implemented. Complexity estimates for both approaches were obtained using 128 training examples were assumed to be used with 8-bit quantization (GMM inputs to fuzzy controller were assumed to have 32-bit quantization due to their resolution requirements) and 10% of the training examples were assumed to be support vectors of the SVM. The relative complexity between the SVM-driven and GMM-driven approach for each of the two stages of the critic-actor control policy are shown in Figure 11. From Figure 11A, it can be seen that at the state estimation stage the SVM-driven approach requires more NOP, with the GMM approach requiring only about 5% SVM NOP. At the state estimation stage, computation in the GMM is dominated by memory while for the SVM it is dominated by NOP. This is because the GMM is a population dependent algorithm, while the SVM only uses the footprint from the population to infer properties. In Figure 11B, the GMM requires a higher NOP for fuzzy inferencing due to its adoption of Gaussian functions as against the triangular function used by the SVM—where triangular COG is simpler to calculate. In terms of memory the GMM requires fewer rules compared to the SVM. It is clear that in the state estimation stage the GMM has less computation and more memory, while at the fuzzy control stage the reverse is the case.

## Discussion

### Critic-Actor Control Policy

The *critic-actor* approach models the relationship between the physician and the automated neuromodulation system. The critic like the “trained clinician” assesses the state of the system based on a cost function (in this case state estimates) and provides the information to the actor. The actor provides control signal based on evaluation from the “informed critic.” In this configuration the state estimator is the critic, while the fuzzy controller is the actor. The main motivation for adopting the critic-actor control policy is because PD suppression can be extremely difficult to achieve due to the limited understanding of the mechanisms underlying PD. This makes it difficult to produce an accurate model that could be used for controller development. It is for this reason that more heuristic methods are proposed. The adaptive scheme exhibits the ability to restore patient LFP characteristics to PD-free conditions for different patients without a change in controller parameters. Changing conditions were monitored through the state estimates, which was the feedback signal. The feedback-loop consists of parkinsonian state (representing symptom severity) determination and stimulation facilitated by the fuzzy controller. The control signal modulates the spectral features to match PD free conditions of each individual patient. The resulting spectral features show that the adaptive scheme has the capacity to restore PD signals to their primary oscillations present under PD-free conditions. More so, using fuzzy inference mechanisms to quantify the dynamics of PD can be very intuitive for modulating therapy. Since it uses rule-based decision making that combines human heuristics into decision making; these rules could be updated into the controller as more knowledge regarding PD is acquired. Effective fuzzy control can only be achieved by adopting the right input pre-processing, in this case state estimates and their rate of change over time were chosen. In the future, external signals e.g., accelerometry activity can be incorporated to produce comprehensive rules that cover an increased number of possible situations. As things stand, optimal control can only be achieved by having a deeper understanding of the underlying mechanisms of DBS and PD—which is more of a clinical challenge. Ultimately, this tool could provide a paradigm on which stimulation can be adapted. The study provides a scheme in which DBS can be adapted using heuristics. To validate the efficacy of the approach, state estimates were obtained using both generative and discriminative machine learning models. Both showed promising results, which are attributable to their self-adjusting nature due to periodic training.

### Model Limitations

At present, a model representing all possible dynamics is far from being realized because there is insufficient knowledge to produce models which closely represent the expected behavior of the system. This is why PD symptom severity is represented by the probability that a patient LFP signal is a PD condition. Apart from clinically sound PD state estimates, several other issues are necessary in order to achieve efficient PD onset control, such as optimal stimulation parameters and how they vary across patients and time. More specifically, the study focused on modulating DBS frequency, it is still under debate which of the parameters (stimulation intensity, pulse width and frequency) is the most beneficial. Nevertheless, controlling one of the parameters could shed more light on how best to control therapy. Currently, a number of assumptions regarding the effect of stimulation on neuronal signals are used to create a stimulation model that draws on the common denominator in all of the theories in Kiss et al. (2002), Carlson et al. (2010), and Chiken and Nambu (2016); which suggest a modulating effect on neuronal signals. This model could be improved if more detailed information on experimental LFP data consisting of stimulation parameters and PD symptom severity are obtained.

Achieving significant progress in aDBS will depend on the correlation between patient state and LFP signal, as well as how stimulation modulates patient LFP. This would require a large set of LFP representing the effect of stimulation on the progression in PD symptoms for a wide range of patients. Presently, the major challenge in adaptive DBS is the difficulty in establishing a direct relationship between patient state and stimulation parameters. This is mainly due to the complexity of post-surgery programming of stimulation parameters by trained clinicians, which can take up to 6 months or more (Bronstein et al., 2011). Because of the limited availability of PD data incorporating the effects of stimulation, stimulation was modeled only by varying stimulation frequencies. This was chosen because stimulation frequency has proven to be more beneficial and reliable than other stimulation parameters (Birdno and Grill, 2008; Baker et al., 2011; Brocker et al., 2013; Su et al., 2018).

Finally, the control policy proposed tends to work better on cases with separable classes and clear states. A summary of the various transition profiles for PD suppression of datasets A to I is presented in the Supplementary Material of the paper. As presented in the Supplementary Material, for non-binary clusters (like the XOR classification problem) or binary clusters with large overlap, additional input information may be required to enable convergence. Convergence of the state estimates to the modal interval of the non-PD state can only be guaranteed for feature spaces with binary clusters and machine learning algorithms that produce an MCC >0.5.

## Conclusion

The work provides theoretical evidence on the possibility of mitigating intractable Parkinsonism by adaptively regulating stimulation using recorded neurophysiological signals. It provides a framework for which if fine-tuned, could lead to the suppression of LFP characteristics in PD patients based on their state estimates (symptom severity) obtained using machine learning algorithms. The dynamic progression of neural signals in PD patients necessitated the adoption of machine learning models for tracking PD. The fuzzy control approach was adopted for computational efficiency and robustness to non-linearity. This was done with hardware implementation in mind, so that the architecture can be deployed in fully implantable aDBS systems that automatically adjust stimulation parameters in real-time in response to changes neurophysiological signals.

## Data Availability Statement

The datasets generated for this study are available on request to the corresponding author.

## Author Contributions

AM performed simulations, analyzed the results, and wrote the manuscript. AD and RB supervised the work and reviewed the manuscript. All authors conceptualized the ideas and approved the manuscript.

## Conflict of Interest

The authors declare that the research was conducted in the absence of any commercial or financial relationships that could be construed as a potential conflict of interest.

## References

[B1] Arlotti M. Marceglia S. Foffani G. Volkmann J. Lozano A. M. Moro E.. (2018). Eight-hours adaptive deep brain stimulation in patients with Parkinson disease. Neurology 90, e971–e976. 10.1212/WNL.0000000000005121 29444973 PMC5858949

[B2] Arlotti M. Rosa M. Marceglia S. Barbieri S. Priori A. (2016). The adaptive deep brain stimulation challenge. Parkinsonism Relat. Disord. 28, 12–17. 10.1016/j.parkreldis.2016.03.020 27079257

[B3] Baizabal-Carvallo J. F. Jankovic J. (2016). Movement disorders induced by deep brain stimulation. Parkinsonism Relat. Disord. 25, 1–9. 10.1016/j.parkreldis.2016.01.014 26806438

[B4] Baker K. B. Zhang J. Vitek J. L. (2011). Pallidal stimulation: effect of pattern and rate on bradykinesia in the non-human primate model of Parkinson's disease. Exp. Neurol. 231, 309–313. 10.1016/j.expneurol.2011.06.012 21767534 PMC3536492

[B5] Barro S. Marin R. (Ed). (2002). Fuzzy Logic in Medicine. Heidelberg: Springer Science and Business Media.

[B6] Belasen A. Rizvi K. Gee L. E. Yeung P. Prusik J. Ramirez-Zamora A.. (2016). Effect of low-frequency deep brain stimulation on sensory thresholds in Parkinson's disease. J. Neurosurg. 126, 397–403. 10.3171/2016.2.JNS152231 27104841

[B7] Birdno M. J. Grill W. M. (2008). Mechanisms of deep brain stimulation in movement disorders as revealed by changes in stimulus frequency. Neurotherapeutics 5, 14–25. 10.1016/j.nurt.2007.10.067 18164480 PMC2200868

[B8] Bologna M. Paparella G. Fasano A. Hallett M. Berardelli A. (2020). Evolving concepts on bradykinesia. Brain 143, 727–750. 10.1093/brain/awz344 31834375 PMC8205506

[B9] Brittain J.-S. Brown P. (2014). Oscillations and the basal ganglia: motor control and beyond. Neuroimage 85(Pt. 2), 637–647. 10.1016/j.neuroimage.2013.05.084 23711535 PMC4813758

[B10] Brocker D. T. Swan B. D. Turner D. A. Gross R. E. Tatter S. B. Koop M. M.. (2013). Improved efficacy of temporally non-regular deep brain stimulation in Parkinson's disease. Exp. Neurol. 239, 60–67. 10.1016/j.expneurol.2012.09.008 23022917 PMC3547657

[B11] Bronstein J. M. Tagliati M. Alterman R. L. Lozano A. M. Volkmann J. Stefani A.. (2011). Deep brain stimulation for Parkinson disease. Arch. Neurol. 68:165. 10.1001/archneurol.2010.260 20937936 PMC4523130

[B12] Brown P. Williams D. (2005). Basal ganglia local field potential activity: character and functional significance in the human. Clin. Neurophysiol. 116, 2510–2519. 10.1016/j.clinph.2005.05.009 16029963

[B13] Carlson J. D. Cleary D. R. Cetas J. S. Heinricher M. M. Burchiel K. J. (2010). Deep brain stimulation does not silence neurons in subthalamic nucleus in Parkinson's patients. J. Neurophysiol. 103, 962–967. 10.1152/jn.00363.2009 19955287 PMC3141810

[B14] Chiken S. Nambu A. (2016). Mechanism of deep brain stimulation: inhibition, excitation, or disruption? Neuroscience 22, 313–322. 10.1177/1073858415581986 PMC4871171 25888630

[B15] Cristianini N. Shawe-Taylor J. (2000). An Introduction to Support Vector Machines and Other Kernel-based Learning Methods. Cambridge: Cambridge University Press.

[B16] Csavoy A. Molnar G. Denison T. (2009). Creating support circuits for the nervous system: considerations for ‘brain-machine' interfacing, in IEEE Symposium on VLSI Circuits (Kyoto), 4–7.

[B17] Dorval A. D. Kuncel A. M. Birdno M. J. Turner D. A. Grill W. M. (2010). Deep brain stimulation alleviates parkinsonian bradykinesia by regularizing pallidal activity. J. Neurophysiol. 104, 911–921. 10.1152/jn.00103.2010 20505125 PMC2934941

[B18] Eusebio A. Thevathasan W. Doyle Gaynor L. Pogosyan A. Bye E. Foltynie T.. (2011). Deep brain stimulation can suppress pathological synchronisation in parkinsonian patients. J. Neurol. Neurosurg. Psychiatry 82, 569–573. 10.1136/jnnp.2010.217489 20935326 PMC3072048

[B19] Feng G. (2006). A survey on analysis and design of model-based fuzzy control systems. IEEE Trans. Fuzzy Syst. 14, 676–697. 10.1109/TFUZZ.2006.883415

[B20] Grant P. F. Lowery M. M. (2013). Simulation of cortico-basal ganglia oscillations and their suppression by closed loop deep brain stimulation. IEEE Trans. Neural Syst. Rehabil. Eng. 21, 584–594. 10.1109/TNSRE.2012.2202403 22695362

[B21] Hacker M. L. Tonascia J. Turchan M. Currie A. Heusinkveld L. Konrad P. E.. (2015). Deep brain stimulation may reduce the relative risk of clinically important worsening in early stage Parkinson's disease. Parkinsonism Relat. Disord. 21, 1177–1183. 10.1016/j.parkreldis.2015.08.008 26306000

[B22] Heida T. Wentink E. C. Marani E. (2013). Power spectral density analysis of physiological, rest and action tremor in Parkinson's disease patients treated with deep brain stimulation. J. Neuroeng. Rehabil. 10:70. 10.1186/1743-0003-10-70 23834737 PMC3722015

[B23] Hell F. Palleis C. Mehrkens J. H. Koeglsperger T. Bötzel K. (2019). Deep brain stimulation programming 2.0: future perspectives for target identification and adaptive closed loop stimulation. Front. Neurol. 10:314. 10.3389/fneur.2019.00314 31001196 PMC6456744

[B24] Johnson A. E. Ghassemi M. M. Nemati S. Niehaus K. E. Clifton D. A. Clifford G. D. (2016). Machine learning and decision support in critical care. Proc. IEEE 104, 444–466. 10.1109/JPROC.2015.2501978 27765959 PMC5066876

[B25] Kiss Z. H. Mooney D. M. Renaud L. Hu B. (2002). Neuronal response to local electrical stimulation in rat thalamus: physiological implications for mechanisms of deep brain stimulation. Neuroscience 113, 137–143. 10.1016/s0306-4522(02)00122-7 12123692

[B26] Kuncel A. M. Grill W. M. (2004). Selection of stimulus parameters for deep brain stimulation. Clin. Neurophysiol. 115, 2431–2441. 10.1016/j.clinph.2004.05.031 15465430

[B27] Little S. Beudel M. Zrinzo L. Foltynie T. Limousin P. Hariz M. (2016). Bilateral adaptive deep brain stimulation is effective in Parkinson's disease*. J. Neurol*. Neurosurg. Psychiatry. 87, 717–721. 10.1136/jnnp-2015-310972 PMC4941128 26424898

[B28] Little S. Brown P. (2012). What brain signals are suitable for feedback control of deep brain stimulation in Parkinson's disease? Ann. N. Y. Acad. Sci. 1, 9–24. 10.1111/j.1749-6632.2012.06650.x PMC3495297 22830645

[B29] Little S. Pogosyan A. Kuhn A. A. Brown P. (2012). β band stability over time correlates with Parkinsonian rigidity and bradykinesia. Exp. Neurol. 236, 383–388. 10.1016/j.expneurol.2012.04.024 22572590 PMC3400051

[B30] Little S. Pogosyan A. Neal S. Zavala B. Zrinzo L. Hariz M.. (2013). Adaptive deep brain stimulation in advanced Parkinson disease. Ann. Neurol. 74, 449–457. 10.1002/ana.23951 23852650 PMC3886292

[B31] Mohammed A. Demosthenous A. (2018). Complementary detection for hardware efficient on-site monitoring of Parkinsonian progress. IEEE J. Emerg. Sel. Top. Circuits Syst. 8, 603–615. 10.1109/JETCAS.2018.2830971

[B32] Mohammed A. Zamani M. Bayford R. Demosthenous A. (2017). Toward on-demand deep brain stimulation using online Parkinson's disease prediction driven by dynamic detection. IEEE Trans. Neural Syst. Rehabil. Eng. 25, 2441–2452. 10.1109/TNSRE.2017.2722986 28682261

[B33] Moreau C. Defebvre L. Destée A. Bleuse S. Clement F. Blatt J. L.. (2008). STN-DBS frequency effects on freezing of gait in advanced Parkinson disease. Neurology 71, 80–84. 10.1212/01.wnl.0000303972.16279.46 18420482

[B34] Moro E. Esselink R. J. Xie J. Hommel M. Benabid A. L. Pollak P. (2002). The impact on Parkinson's disease of electrical parameter settings in STN stimulation. Neurology 59, 706–713. 10.1212/wnl.59.5.706 12221161

[B35] Passino K. Yurkovich S. (1998). Fuzzy Control, 1st Edn. Boston, MA: Addison Wesley Publishing Company.

[B36] Picillo M. Lozano A. M. Kou N. Puppi Munhoz R. Fasano A. (2016). Programming deep brain stimulation for parkinson's disease: the Toronto western hospital algorithms. Brain Stimul. 9, 425–437. 10.1016/j.brs.2016.02.004 26968806

[B37] Prandoni P. Vetterli M. (2008). Signal Processing for Communications, 1t Edn. Boca Raton, FL: CRC Press.

[B38] Priori A. Foffani G. Rossi L. Marceglia S. (2012). Adaptive deep brain stimulation (aDBS) controlled by local field potential oscillations. Exp. Neurol. 245, 77–86. 10.1016/j.expneurol.2012.09.013 23022916

[B39] Rossow A. B. Salles E. O. T. Coco K. F. (2011). Automatic sleep staging using a single-channel EEG modeling by Kalman filter and HMM, in ISSNIP Biosignals and Biorobotics Conference (Vitoria), 1–6.

[B40] Sajda P. (2006). Machine learning for detection and diagnosis of disease. Annu. Rev. Biomed. Eng. 8, 537–565. 10.1146/annurev.bioeng.8.061505.095802 16834566

[B41] Soltesz K. Hahn J. O. Hägglund T. Dumont G. A. Ansermino J. (2013). Individualized closed-loop control of propofol anesthesia: a preliminary study. Biomed. Signal Process. Control. 8, 500–508. 10.1016/j.bspc.2013.04.005

[B42] Su D. Chen H. Hu W. Liu Y. Wang Z. Wang X.. (2018). Frequency-dependent effects of subthalamic deep brain stimulation on motor symptoms in Parkinson's disease: a meta-analysis of controlled trials. Sci. Rep. 8, 1–9. 10.1038/s41598-018-32161-3 30262859 PMC6160461

[B43] Vercruysse S. Vandenberghe W. Münks L. Nuttin B. Devos H. Nieuwboer A. (2014). Effects of deep brain stimulation of the subthalamic nucleus on freezing of gait in Parkinson's disease: a prospective controlled study. J. Neurol. Neurosurg. Psychiatry 85, 871–877. 10.1136/jnnp-2013-306336 24396010

[B44] Whitmer D. de Solages C. Hill B. Yu H. Henderson J. M. Bronte-Stewart H. (2012). High frequency deep brain stimulation attenuates subthalamic and cortical rhythms in Parkinson's disease. Front. Hum. Neurosci. 6:155. 10.3389/fnhum.2012.00155 22675296 PMC3366347

[B45] Wu D. Lance B. J. Lawhern V. J. (2017). Guest editorial for the special section on brain computer interface (BCI). IEEE Trans. Fuzzy Syst. 25, 1–2. 10.1109/TFUZZ.2017.2652799

[B46] Xie T. Kang U. J. Warnke P. (2012). Effect of stimulation frequency on immediate freezing of gait in newly activated STN DBS in Parkinson's disease. J. Neurol. Neurosurg. Psychiatry 83, 1015–1017. 10.1136/jnnp-2011-302091 22696586

[B47] Zarkogianni K. Vazeou A. Mougiakakou S. G. Prountzou A. Nikita K. S. (2011). An insulin infusion advisory system based on autotuning nonlinear model-predictive control. IEEE Trans. Biomed. Eng. 58, 2467–2477. 10.1109/TBME.2011.2157823 21622071

[B48] Zavitsanou S. Chakrabarty A. Dassau E. Doyle F. (2016). Embedded control in wearable medical devices: application to the artificial pancreas. Processes 4:35 10.3390/pr4040035

