# Supplementary material for: A Framework for Adapting Deep Brain Stimulation Using Parkinsonian State Estimates
Source: Front Neurosci. 2020 May 19;14:499. doi: 10.3389/fnins.2020.00499 (PMC7248244; doi:10.3389/fnins.2020.00499)
Supplement: Supplementary file 1 [file Data_Sheet_1.pdf]

Supplementary Material

Using SVM and GMM driven approaches, the various profiles for PD suppression are depicted in **Figures S1–S9** for datasets A to I respectively. **Figures S1A–S9A** depict the profile for the SVM driven approach, while **Figures S1B–S9B** represent that of the GMM driven approach. The SVM driven approach works very well for all datasets except dataset G, which it found difficult to identify its settling (modal) state as a result of the high overlap between states. For the GMM driven approach, it did not work very well for datasets A and G. This is also as a result of the high overlap between the PD and non-PD clusters. The state transition profile in **Figures S1–S9** demonstrates that the SVM performs better than the GMM in identifying and settling at the modal state. This corroborates the information in the manuscript for accuracy and correlation measurements.

It is clear that the control policy proposed tends to work better on cases with separable classes and clear states. For non-binary clusters (like the XOR classification problem) or binary clusters with large overlap like **Figures S1,S7** (a hypothetical case is also shown in **Figure S10**), additional input information may be required to enable convergence. In **Figure S10**, the interquartile range which covers the modal class for both PD and non-PD clusters have near complete overlap. This will make fitting a machine learning model very difficult. Using this control policy, convergence of the state estimates to the modal interval of the non-PD state can only be guaranteed for feature spaces with binary clusters and machine learning algorithms that produce an MCC greater than 0.5.

From the forgoing, it is clear that both (SVM and GMM driven) machine learning configurations achieve PD suppression to the desired state in at least seven out of nine cases.

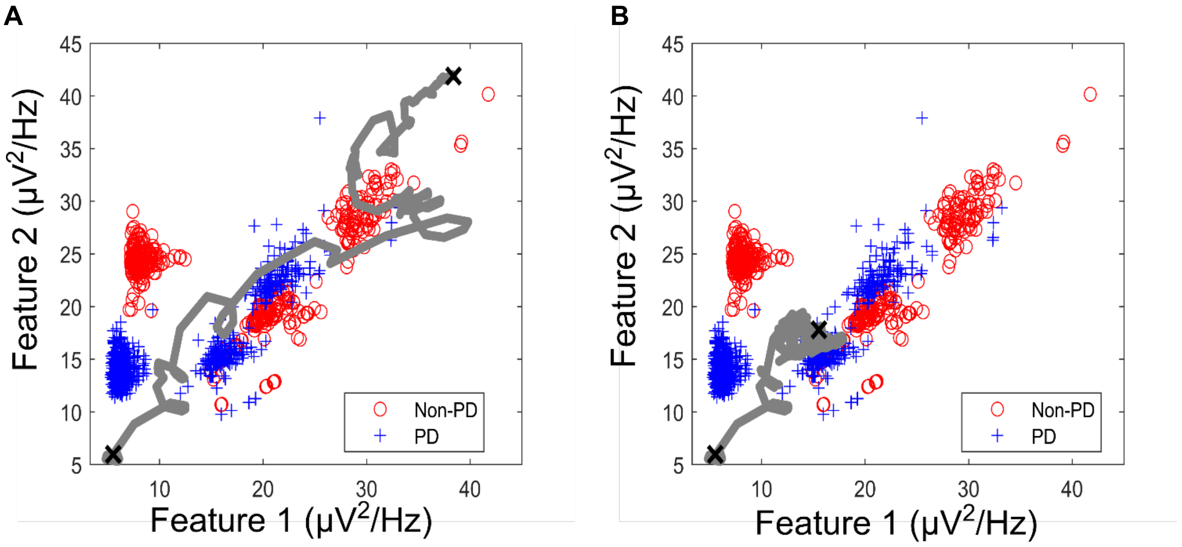

Figure S1 | State transition of PD suppression on feature space of patient/dataset A. **(A)** Showing PD state profile on a feature space using SVM for state estimation, with "X" markers showing start (from PD) and settling (non-PD) positions. The feature space trajectory is indicated in grey. **(B)** Showing PD state transition on a feature space using GMM for state estimation, with "X" markers showing start (from PD) and settling (non-PD) positions. The feature space trajectory is indicated in grey.

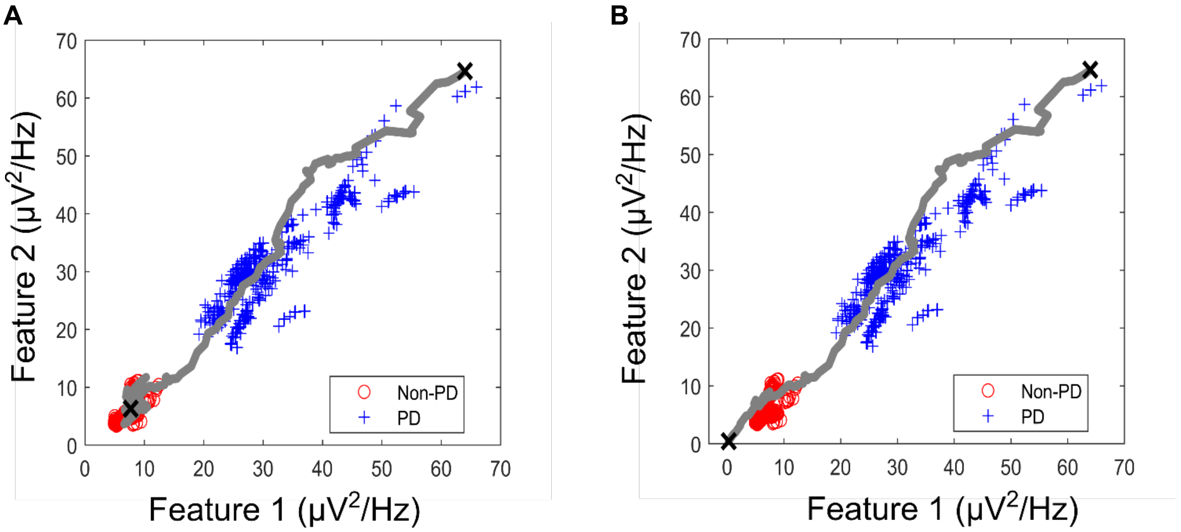

Figure S2 | State transition of PD suppression on feature space of patient/dataset B. **(A)** Showing PD state profile on a feature space using SVM for state estimation, with "X" markers showing start (from PD) and settling (non-PD) positions. The feature space trajectory is indicated in grey. **(B)** Showing PD state transition on a feature space using GMM for state estimation, with "X" markers showing start (from PD) and settling (non-PD) positions. The feature space trajectory is indicated in grey.

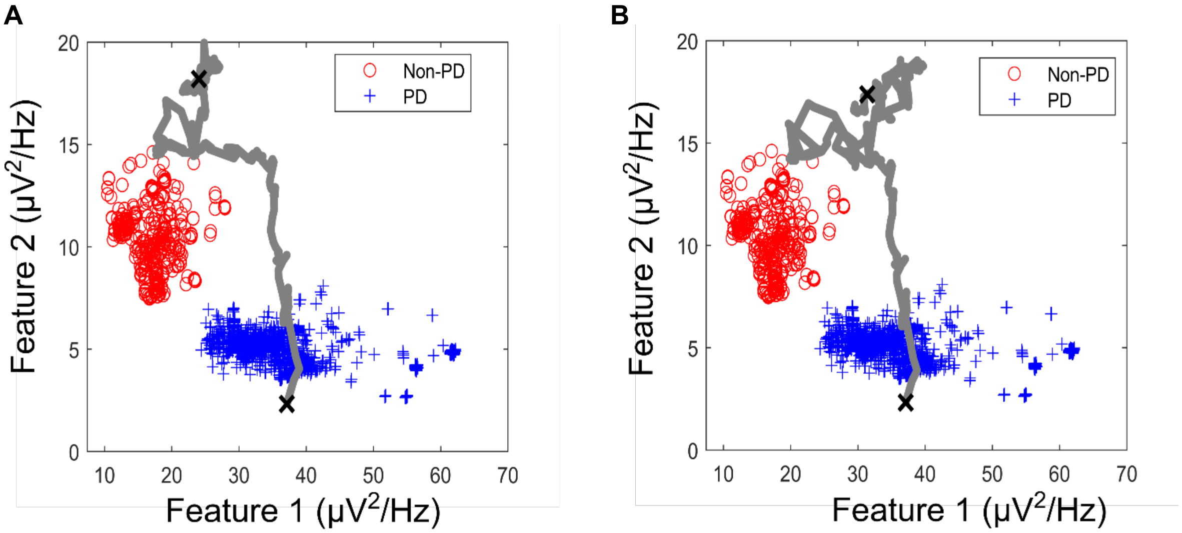

Figure S3 | State transition of PD suppression on feature space of patient/dataset C. **(A)** Showing PD state profile on a feature space using SVM for state estimation, with "X" markers showing start (from PD) and settling (non-PD) positions. The feature space trajectory is indicated in grey. **(B)** Showing PD state transition on a feature space using GMM for state estimation, with "X" markers showing start (from PD) and settling (non-PD) positions. The feature space trajectory is indicated in grey.

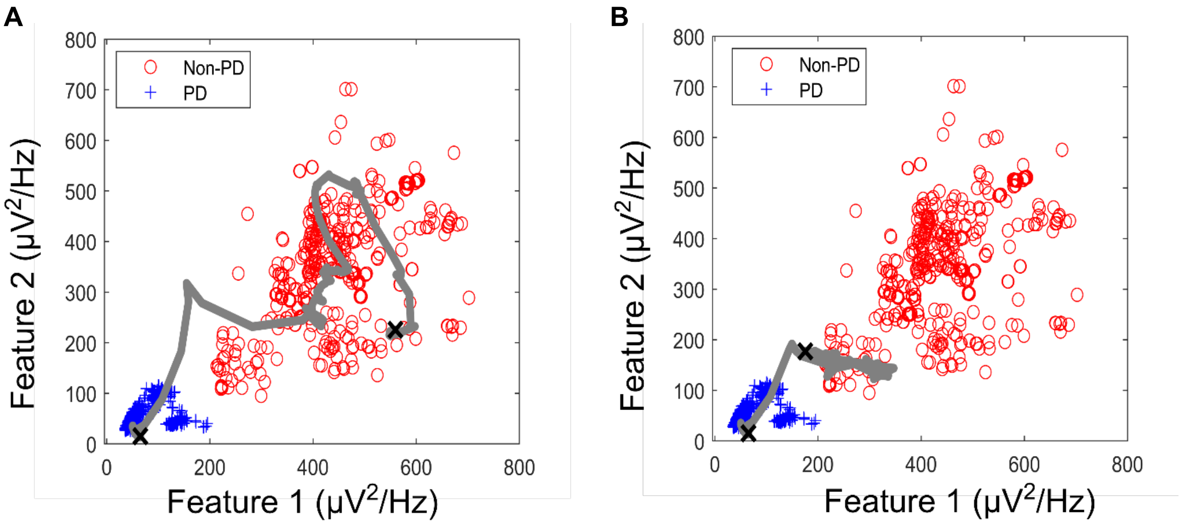

Figure S4 | State transition of PD suppression on feature space of patient/dataset D. **(A)** Showing PD state profile on a feature space using SVM for state estimation, with "X" markers showing start (from PD) and settling (non-PD) positions. The feature space trajectory is indicated in grey. **(B)** Showing PD state transition on a feature space using GMM for state estimation, with "X" markers showing start (from PD) and settling (non-PD) positions. The feature space trajectory is indicated in grey.

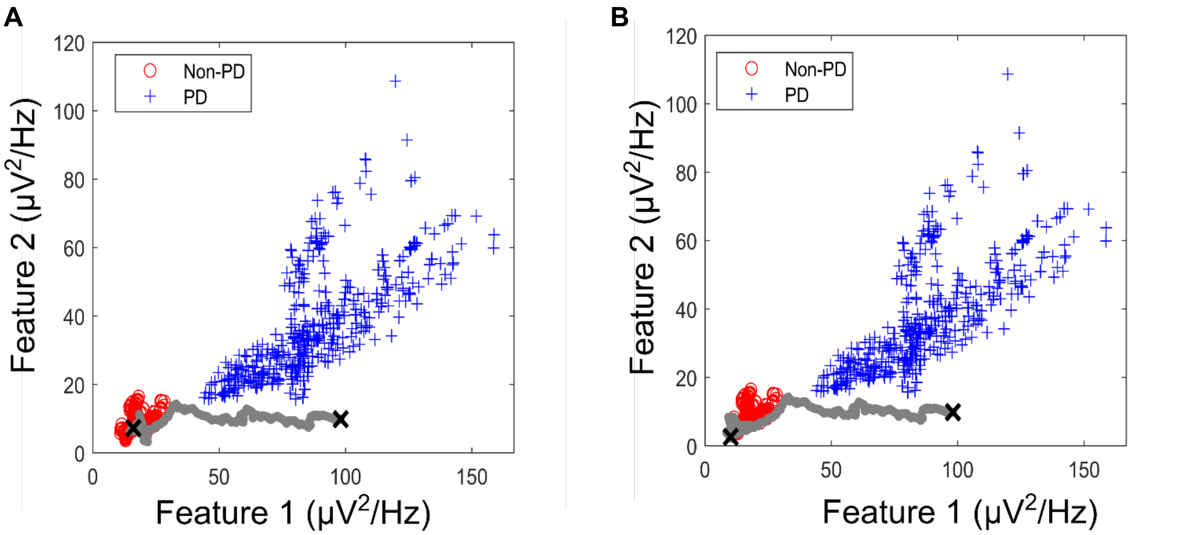

Figure S5 | State transition of PD suppression on feature space of patient/dataset E. **(A)** Showing PD state profile on a feature space using SVM for state estimation, with "X" markers showing start (from PD) and settling (non-PD) positions. The feature space trajectory is indicated in grey. **(B)** Showing PD state transition on a feature space using GMM for state estimation, with "X" markers showing start (from PD) and settling (non-PD) positions. The feature space trajectory is indicated in grey.

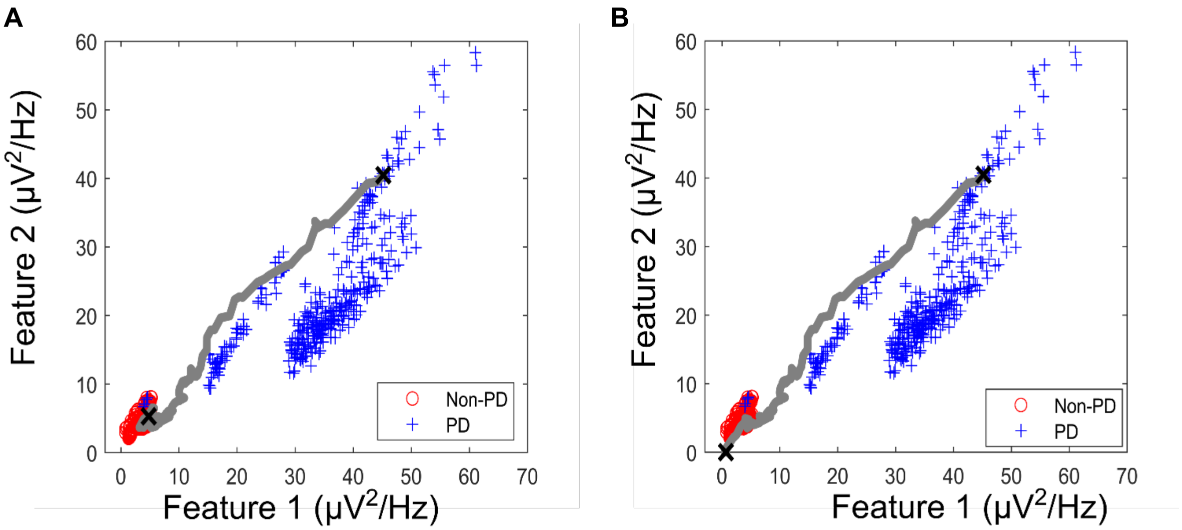

Figure S6 | State transition of PD suppression on feature space of patient/dataset F. **(A)** Showing PD state profile on a feature space using SVM for state estimation, with "X" markers showing start (from PD) and settling (non-PD) positions. The feature space trajectory is indicated in grey. **(B)** Showing PD state transition on a feature space using GMM for state estimation, with "X" markers showing start (from PD) and settling (non-PD) positions. The feature space trajectory is indicated in grey.

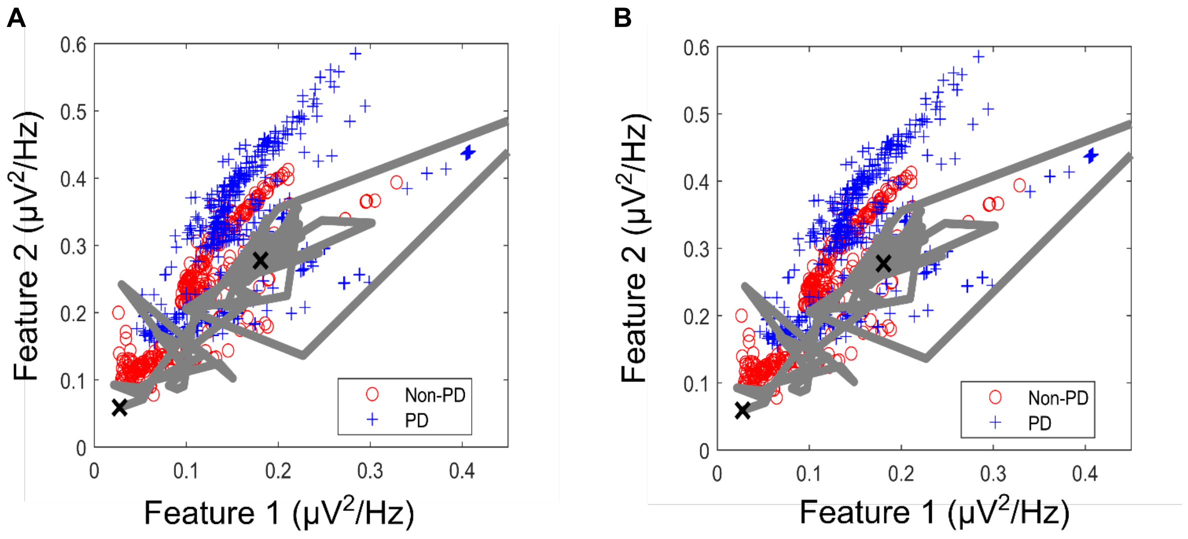

Figure S7 | State transition of PD suppression on feature space of patient/dataset G. **(A)** Showing PD state profile on a feature space using SVM for state estimation, with "X" markers showing start (from PD) and settling (non-PD) positions. The feature space trajectory is indicated in grey. **(B)** Showing PD state transition on a feature space using GMM for state estimation, with "X" markers showing start (from PD) and settling (non-PD) positions. The feature space trajectory is indicated in grey.

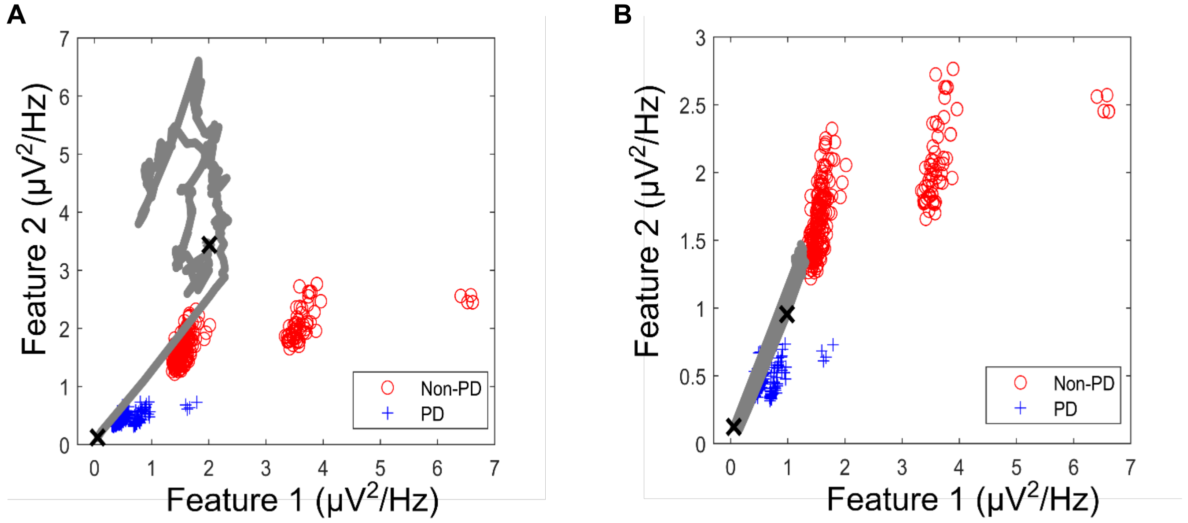

Figure S8 | State transition of PD suppression on feature space of patient/dataset H. **(A)** Showing PD state profile on a feature space using SVM for state estimation, with "X" markers showing start (from PD) and settling (non-PD) positions. The feature space trajectory is indicated in grey. **(B)** Showing PD state transition on a feature space using GMM for state estimation, with "X" markers showing start (from PD) and settling (non-PD) positions. The feature space trajectory is indicated in grey.

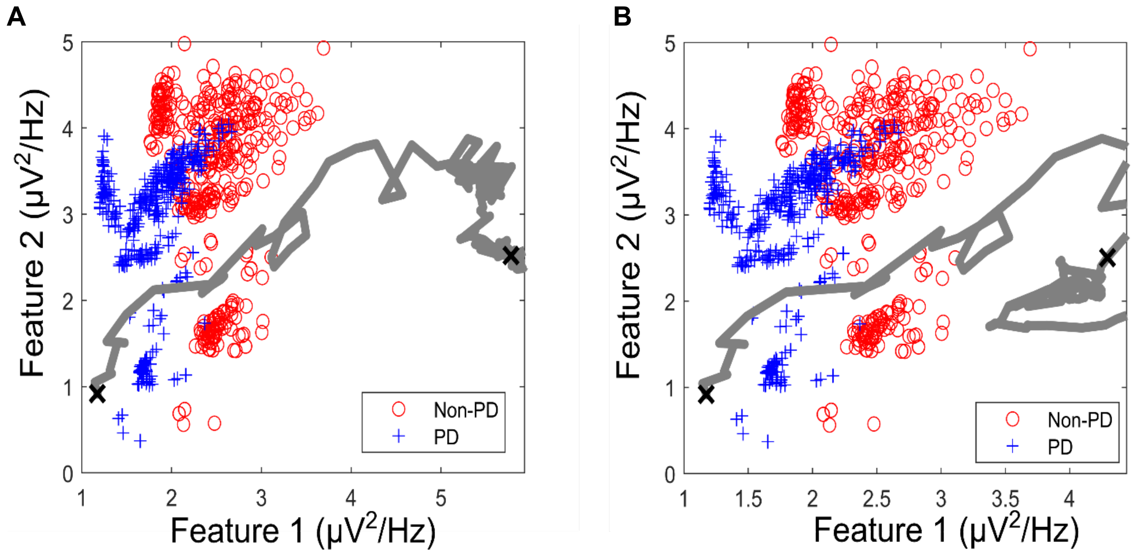

Figure S9 | State transition of PD suppression on feature space of patient/dataset I. **(A)** Showing PD state profile on a feature space using SVM for state estimation, with "X" markers showing start (from PD) and settling (non-PD) positions. The feature space trajectory is indicated in grey. **(B)** Showing PD state transition on a feature space using GMM for state estimation, with "X" markers showing start (from PD) and settling (non-PD) positions. The feature space trajectory is indicated in grey.

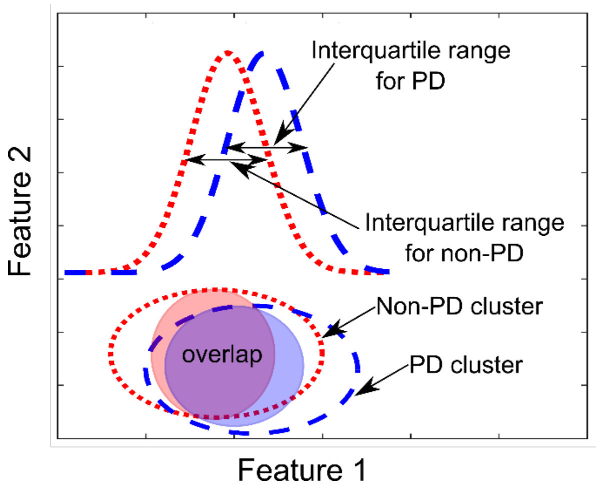

Figure S10 | 2-D representation of feature space with a very high similarity between PD and non-PD clusters. It has a near complete overlap in the training examples that fall within the interquartile ranges of both clusters. The interquartile range of the non-PD cluster is represented by the light-red circle inscribed in the non-PD cluster, and also indicated is the PDF for the non-PD cluster. While that of PD is the light-blue circle inscribed in the PD cluster, and also indicated is the PDF for the PD cluster.
